# Glucocorticoids Enhance Muscle Proteolysis through a Myostatin-Dependent Pathway at the Early Stage

**DOI:** 10.1371/journal.pone.0156225

**Published:** 2016-05-26

**Authors:** Ruxia Wang, Hongchao Jiao, Jingpeng Zhao, Xiaojuan Wang, Hai Lin

**Affiliations:** Department of Animal Science, Shandong Agricultural University, Shandong Key Lab for Animal Biotechnology and Disease Control, Taian, Shandong, 271018, P. R. China; Institut de Myologie, FRANCE

## Abstract

Myostatin, a member of the TGF-β superfamily of secreted proteins, is expressed primarily in skeletal muscle. It negatively regulates muscle mass and is associated with glucocorticoid-induced muscle atrophy. However, it remains unclear whether myostatin is involved in glucocorticoid-induced muscle protein turnover. The aim of the present study was to investigate the role of myostatin in protein metabolism during dexamethasone (DEX) treatment. Protein synthesis rates and the expression of the genes for myostatin, ubiquitin-proteasome atrogin-1, MuRF1, FoxO1/3a and mTOR/p70S6K were determined. The results show that DEX decreased (*P*<0.05) protein synthesis rates while increasing the abundance of myostatin. DEX increased (*P*<0.05) the level of phospho-FoxO1/3a (Thr 24/32) and the expression of MuRF1. In contrast, DEX treatment had no detectable effect on atrogin-1 protein levels (*P*>0.05). The phosphorylation levels of mTOR and p70S6K were decreased by DEX treatment (*P*<0.05). Follistatin treatment inhibited the DEX-induced increase in myostatin (*P*<0.05) and the activation of phosphor-FoxO1/3a (Thr 24/32) (*P*< 0.05) and MuRF1 (*P*<0.05). Follistatin treatment had no influence on the protein synthesis rate or on the phosphorylation levels of mTOR (Ser 2448) and p70S6K (Thr 389) (*P*> 0.05). In conclusion, the present study suggests that the myostatin signalling pathway is associated with glucocorticoid-induced muscle protein catabolism at the beginning of exposure. Myostatin is not a main pathway associated with the suppression of muscle protein synthesis by glucocorticoids.

## Introduction

Stress can cause muscle wasting and atrophy [1, 2]. When animals suffer from severe stress, excessive glucocorticoids in the circulation cause muscle loss by enhancing proteolysis and impeding protein synthesis [3, 4, 5]. The increased protein breakdown during stress is mainly caused by the activation of proteolytic systems, such as the Ca2+-dependent and the ATP-ubiquitin-dependent systems [6]. In particular, the ubiquitin-proteasome system seems to play a major role in the catabolic action of glucocorticoids [7]. Two important muscle-specific ubiquitin E3 ligases, muscle-specific RING finger protein 1 (MuRF1) and atrogin-1, have been shown to be related to a variety of atrophic conditions.

Myogenic differentiation is a complex and well-coordinated process that is regulated by growth factors, principally myostatin and insulin-like growth factor-I (IGF-I). Myostatin is a member of the transforming growth factor β (TGF-β) family and acts as a negative regulator of skeletal muscle growth. Myostatin is essential for the negative regulation of skeletal muscle growth [8]. A mutation in the coding region of myostatin results in a double-muscling phenotype in Belgian blue and Piedmontese cattle [9, 10]. The mutation of myostatin is associated with muscle hypertrophy in humans as well [11]. A blockade of endogenous myostatin results in an adverse increase in muscle mass and size and a decrease in muscle degeneration in an X chromosome-linked muscular dystrophy (mdx) mouse-model of Duchenne muscular dystrophy [12]. Knockout of the myostatin gene induces a significant increase in muscle mass in mice after developmental muscle growth has ceased [13]. As a negative regulator of myogenesis, myostatin is involved in the control of myoblast proliferation. In C2C12 muscle cells, recombinant myostatin proteins inhibit cell proliferation, DNA synthesis, and protein synthesis, suggesting that myostatin may control muscle mass by inhibiting muscle growth or regeneration [14]. Myostatin inhibits myoblast differentiation through an interaction with Smad 3 [15]. The negative effect of myostatin on muscle growth is counteracted by the positive effect of insulin-like growth factor-1 (IGF-I), which promotes protein synthesis via activation of the phosphoinositide 3-kinase (PI3K)/Akt signalling pathway [16]. The IGF-I/PI3K/Akt pathway may inhibit myostatin signalling during myoblast differentiation [17].

Myostatin has been implicated in several forms of muscle wasting, including the severe cachexia observed as a result of conditions such as AIDS and liver cirrhosis. Myostatin increases the activity of the ubiquitin-proteasome system and negatively regulates the activity of the Akt pathway, thereby inducing muscle atrophy [18]. Myostatin results in an increased activation of atrogin-1 and MuRF1 [19, 20]. Moreover, the overexpression of myostatin contributes to muscle atrophy under several conditions, particularly in the presence of glucocorticoids [2, 21, 22]. Myostatin has been shown to reverse the IGF-I/PI3K/AKT hypertrophy pathway by inhibiting AKT phosphorylation, thereby increasing the levels of active FoxO1 and allowing for an increased expression of atrophy-related genes [19]. Hence, we hypothesized that myostatin is involved in the muscle atrophy induced by glucocorticoids.

In the present study, the role of myostatin on the protein synthesis of C2C12 cells in the presence of dexamethasone (DEX), a synthetic glucocorticoid, was investigated *in vitro*. The protein synthesis rate was estimated using a nonradioactive method by labelling the newly synthesized polypeptides with low concentrations of puromycin and subsequently detecting these proteins with an anti-puromycin antibody [23, 24]. The involvement of the ubiquitin-proteasome and mTOR/p70S6K signalling pathways were also evaluated in this study.

## Materials and Methods

### Cell culture

Murine C2C12 skeletal myoblasts (CCTCC, China) were seeded in 6-well plates and cultured in high-glucose Dulbecco’s Modified Eagle’s Medium (DMEM; HyClone, China) supplemented with 10% FBS (Gibco, USA), 100 U/mL penicillin, and 0.1 mg/mL streptomycin (Solarbio, China) and maintained at 37°C in a humidified atmosphere with 5% CO_2_. The medium was changed every two days. At 80% confluence, the cells were incubated with DMEM containing 2% horse serum (Gibco, USA) to induce myotube formation. After 84 h of differentiation, the cultures were shifted to serum-free DMEM for 12 hours and the C2C12 cells were prepared for treatment with agents as described below.

### DEX treatment

The C2C12 cells were incubated in DMEM supplemented with 100 μM dexamethasone (DEX; Cisen, China) for different time periods (12 h, 24 h, 36 h, 48 h) [25]. The level of myostatin expression was estimated using real-time PCR and western blot methods.

### Follistatin treatment

To investigate the role of myostatin in glucocorticoid-induced catabolism, follistatin, a powerful antagonist of myostatin, was used to inhibit the influence of myostatin [26]. The C2C12 cells were randomly subjected to the following treatments: DEX (100 μM), follistatin (800 ng/ml; Sigma, USA), DEX (100 μM) plus follistatin (800 ng/mL), and control (no treatment). After a 36-hour treatment, the cultures were supplemented with puromycin (1 μM; Solarbio, China) for another 30 min, and the cells were then harvested for further analysis.

### Measurement of protein synthesis rate

The protein synthesis rate was determined using a nonradioactive method [23]. The newly synthesized polypeptides were labelled with low concentrations of puromycin, and these proteins were subsequently detected with an anti-puromycin antibody. The accumulation of puromycin-conjugated peptides into nascent peptide chains reflects the rate of protein synthesis [23, 24].

### RNA extraction and quantitative real-time PCR analyses

RNA was extracted using TRIzol (Invitrogen, USA). The RNA concentration was measured by spectrophotometry (Eppendorf, Germany), and the purity of the RNA was verified by calculating the ratio between the absorbance values at 260 and 280 nm (A260/280 ≈1.75–2.01). Total RNA (1 μg) was used for first-strand cDNA synthesis with the Prime Script^TM^ RT reagent kit for RT-PCR (TaKaRa, China) according to the manufacturer's instructions. Then, real-time PCR was performed using SYBR Premix Ex Taq^TM^ (TaKaRa, China). Following the manufacturer’s protocol, the resulting cDNA was amplified in a 20-μL PCR reaction system containing 0.2 μmol/L of each specific primer (Sangon, China) and of the SYBR Green master mix. An ABI 7500 PCR machine (Applied Biosystems; Thermo, USA) was used for the amplification of cDNA, and primers were designed with Primer 6.0 software. The primers were as follows: mouse myostatin: forward, CAGGAGAAGATGGGCTGAATCC and reverse, AAGCCCAAAGTCTCTCCGGG; atrogin-1: forward, GCTGGATTGGAAGAAGAT and reverse, GAGAATGTGGCAGTGTTTG; MuRF1: forward, CTGGAGGTCGTTTCCGTTGC and reverse, TCGGGTGGCTGCCTTTCTGC; FoxO3: forward, CCCTAACCCAGCAGAGACTGT and reverse, GGAAACAAACACAAGACGACACT; FoxO1: forward, GAGTGGATGGTGAAGAGCGT and reverse, GGGACAGATTGTGGCGAAT; IGF-I: forward, ATGCTCTTCAGTTCGTGTGTGG and reverse, TCTTGGGCATGTCAGTGTGG; and β-actin: forward, ACCACACCTTCTACAATGAG and reverse, ACGACCAGAGGCATACAG. As a control, β-actin primers were used in a duplexed reaction, and all of the mRNA values were normalised to the β-actin values.

### Protein preparation and western blot analyses

C2C12 myotubes were homogenised in 0.2 mL of lysis buffer (Beyotime, China) and kept on ice during the trial procedure. The homogenate was centrifuged at 12,000 *g* for 5 min at 4°C, and the supernatant was collected. Protein concentration was assayed using a BCA assay kit (Beyotime, China) according to the manufacturer’s protocol. Aliquots of 18 μg of protein were separated with 7.5–10% SDS polyacrylamide gels (Bio-Rad, Richmond, 246 CA) according to the method described by Laemmli (1970) [27], and the proteins were then transferred onto a PVDF membrane (Millipore, USA) at 200 mA for 2 h in a Tris-glycine buffer with 20% anhydrous ethanol at 4°C. Membranes were blocked with western blocking buffer (Beyotime, China) for 1 h at room temperature. The membranes were then probed with primary antibodies at 4°C with gentle shaking overnight. The primary antibodies used were anti-myostatin, anti-MuRF1, anti-atrogin-1, anti-phospho-FoxO1/3a (Thr^24/32^) (Abcam, UK), anti-FoxO1, anti-mTOR, anti-phospho-mTOR (Cell Signalling Technologies, USA), anti-mouse puromycin (Kerafast, USA), and anti-β-actin (Beyotime, China). After being washed, the membranes were incubated with horseradish peroxidase-linked anti-rabbit, anti-mouse, or anti-rat secondary antibodies for 4 h at 4°C. Membranes were then visualized by exposure to Hyperfilm ECL (Beyotime, China). Films were scanned, and specific bands were quantified using ImageJ 1.43 software (National Institutes of Health, USA). The band intensity was normalised to the β-actin band in the same sample.

### Statistical analysis

The main effect of each treatment on protein metabolism was evaluated using a one-way ANOVA performed with the Statistical Analysis Systems statistical software package (Version 8e, SAS Institute, USA). Multiple comparisons between the means were conducted using Duncan’s honestly significant difference test. The means were considered to be significantly different at *P*< 0.05.

## Results

### Effect of DEX treatment

**Fig 1:**
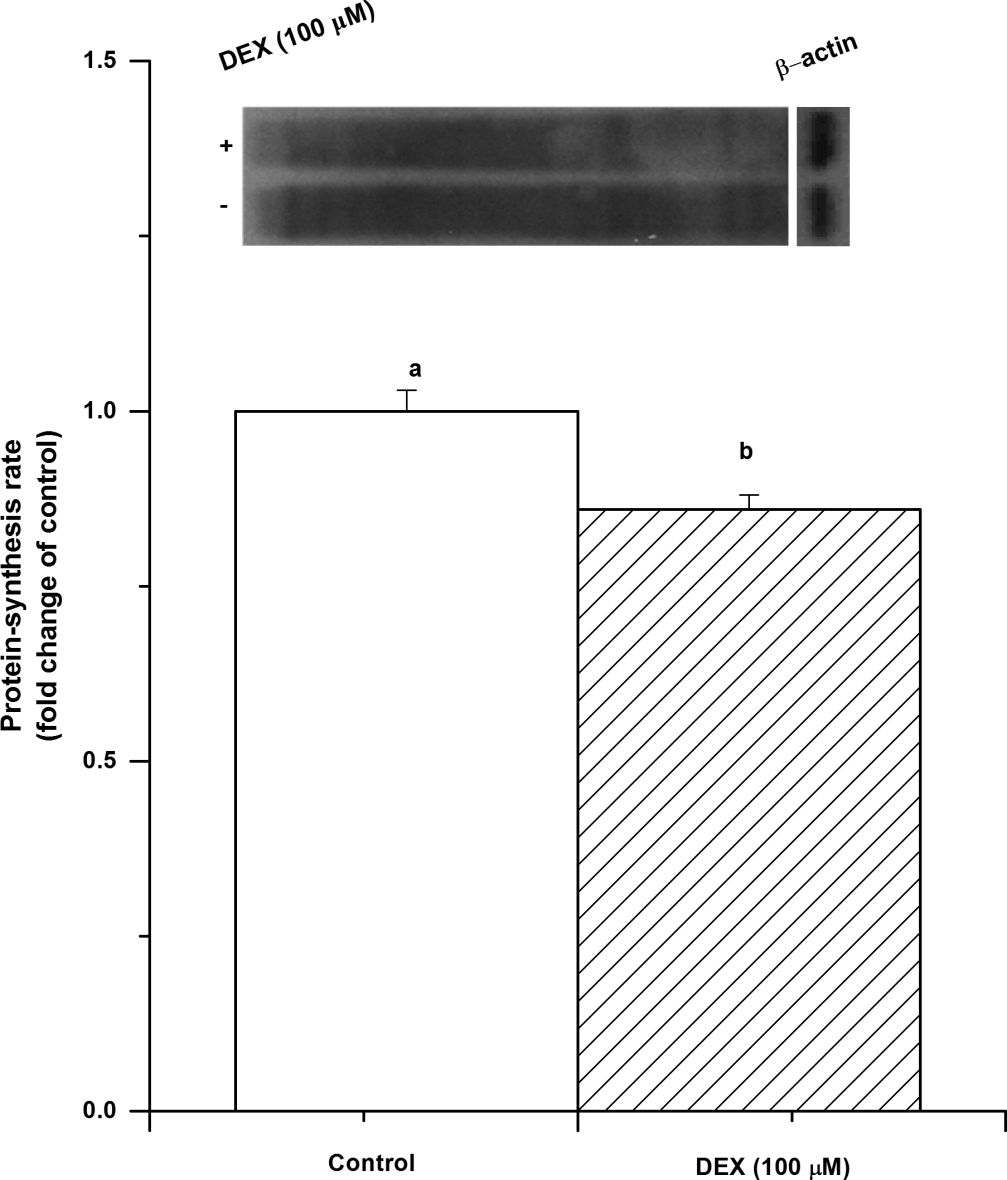
The inhibitory effect of dexamethasone (DEX) on protein synthesis. After DEX (100 μM) treatment for 36 h, C2C12 cells are cultured with puromycin (1 μM) for extra 30 min and then the protein-synthesis rate was assayed by western blot. The values are presented as the means ± SEM (n = 6). ^a,b^ Means with different letters differ significantly (*P <* 0.05).

**Fig 2:**
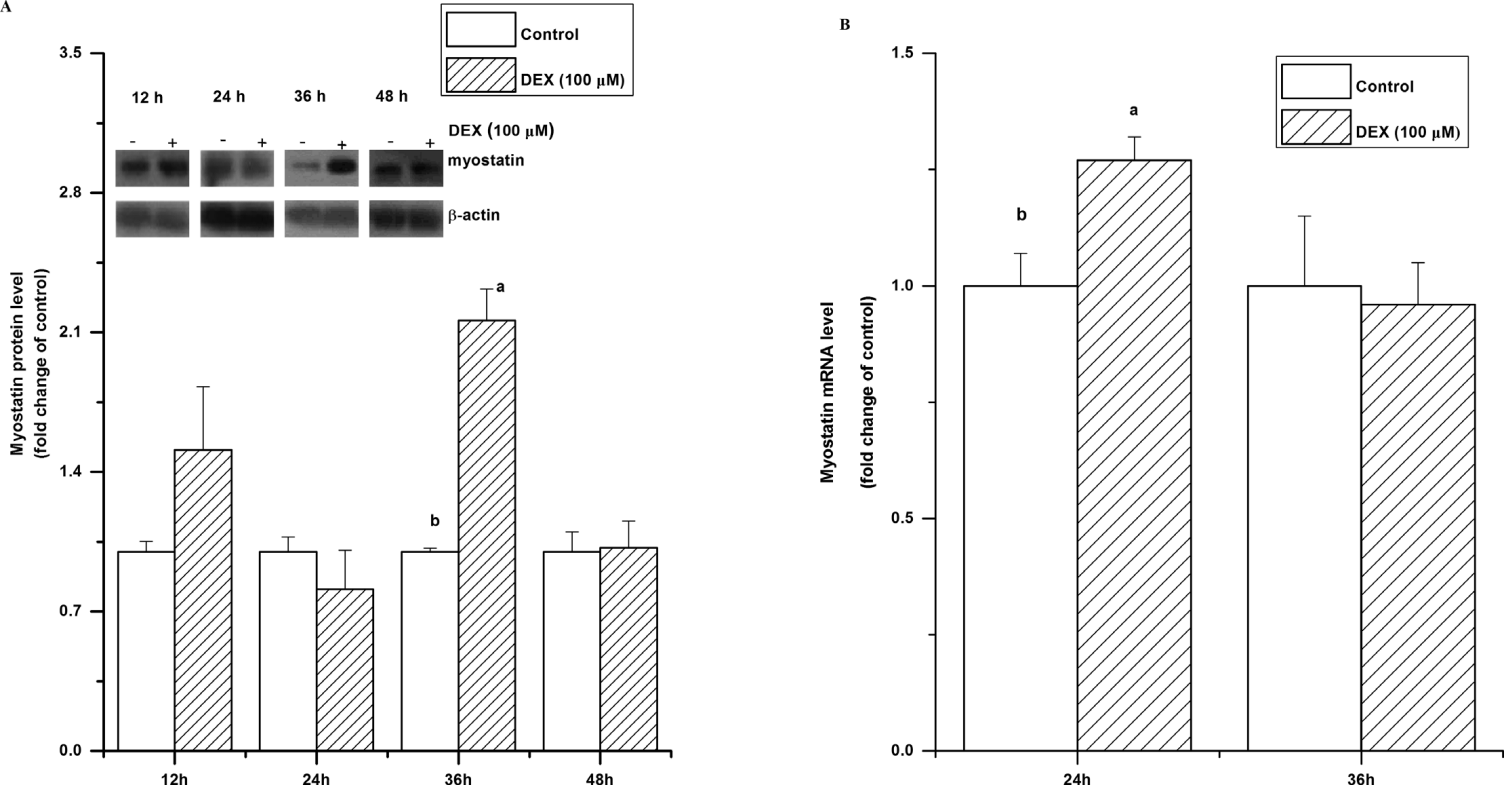
Effect of dexamethasone (DEX) on myostatin expression. Myostatin protein levels (A) and mRNA levels (B) in C2C12 cells treated with dexamethasone (DEX, 100 μM) for 12 h, 24 h, 36 h and 48 h. The values are presented as the means ± SEM (n = 6). ^a,b^ Means with different letters differ significantly (*P <* 0.05).

The protein synthesis rate was significantly decreased by DEX treatment (*P*<0.05, Fig 1). However, the level of myostatin protein was significantly increased (*P*<0.05) by DEX treatment at 36 h, while there was no significant change at 24 and 48 h (*P*>0.05, Fig 2A). The level of myostatin mRNA was significantly upregulated by DEX after a 24 h treatment (*P*<0.05) but not after 36 h (*P*>0.05, Fig 2B).

**Fig 3:**
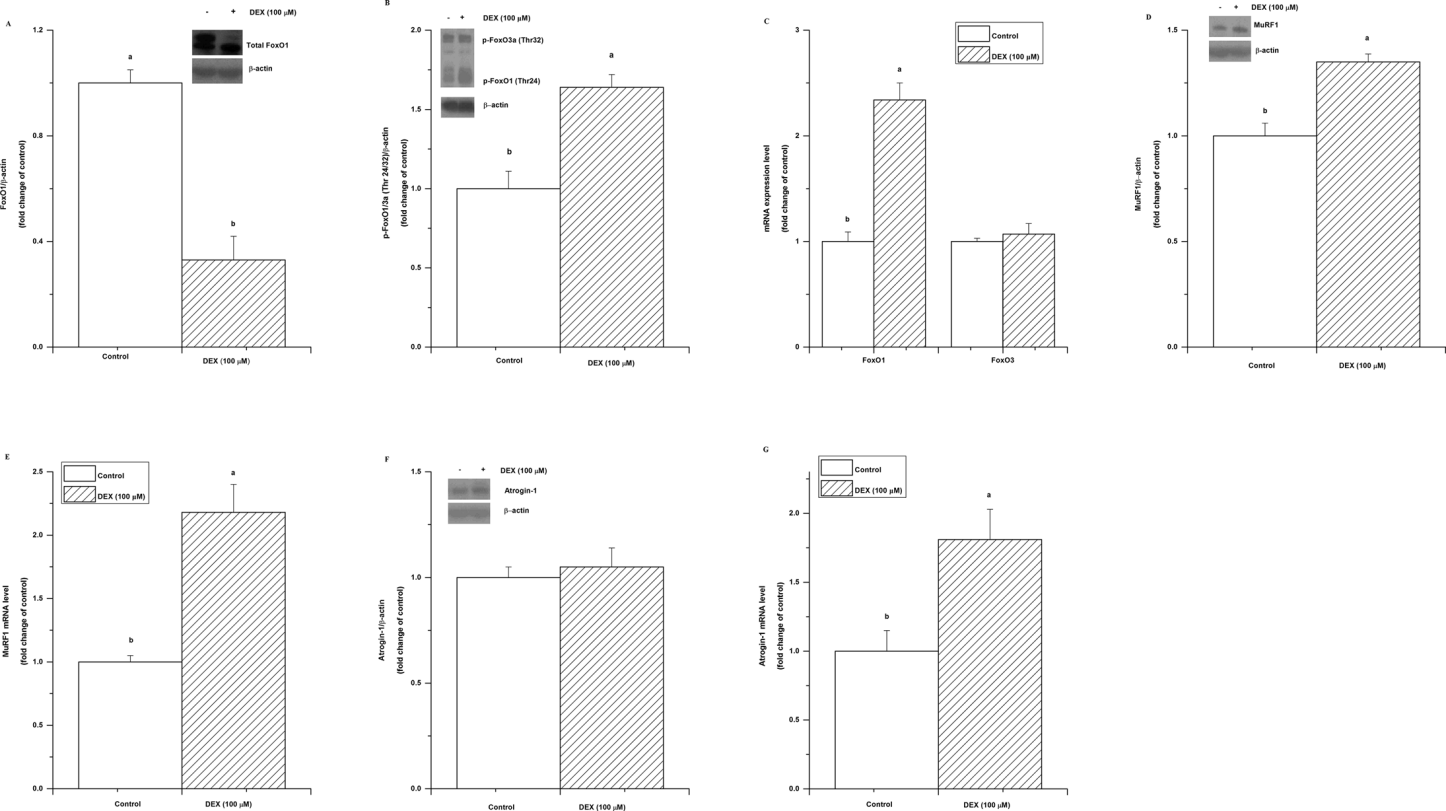
Effect of dexamethasone (DEX) treatment on the expression of ubiquitin-proteasome-related factors involved with protein catabolism. The total FoxO1 protein (A), phospho-FoxO1/3a (Thr 24/32) (B), FoxO1 and FoxO3 mRNA levels (C), MuRF1 protein (D), MuRF1 mRNA (E), atrogin-1 protein (F), and atrogin-1 mRNA (G) in C2C12 cells treated with DEX (100 μM) for 36 h. The values are presented as the means ± SEM (n = 6). ^a,b^ Means with different letters differ significantly (*P <* 0.05).

The effect of DEX on the ubiquitin-proteasome pathway was investigated. DEX treatment significantly decreased the level of the protein FoxO1 (*P*<0.05, Fig 3A) but increased the phosphor-FoxO1/3a (Thr 24/32) level (*P*<0.05, Fig 3B). DEX upregulated the transcription of FoxO1 mRNA (*P*< 0.05), but it had no influence on the mRNA level of FoxO3 (*P*>0.05, Fig 3C).

The two downstream proteins of FoxO1, MuRF1 and atrogin-1, were then measured. DEX treatment dramatically increased the expression of MuRF1 at both the protein (*P*< 0.05, Fig 3D) and mRNA levels (*P*< 0.05, Fig 3E). In contrast, DEX treatment significantly upregulated (*P*<0.05, Fig 3F) the mRNA level of atrogin-1 but had no detectable effect on the protein level of atrogin-1 (*P*>0.05, Fig 3G).

**Fig 4:**
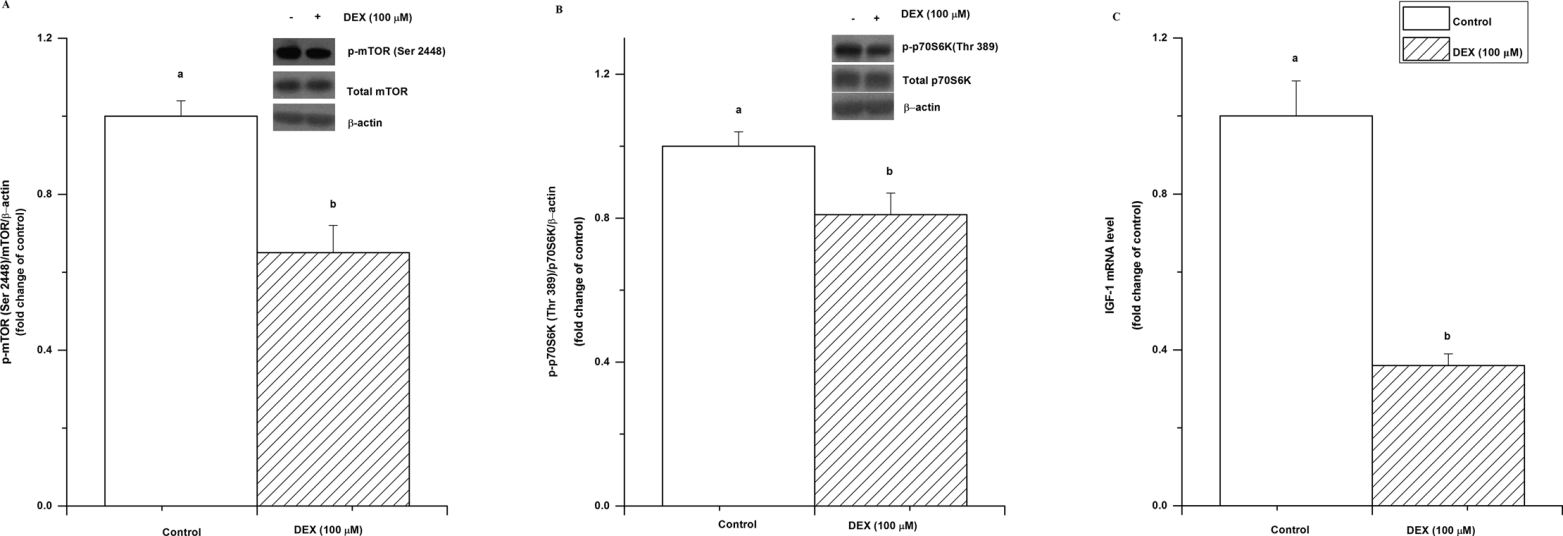
Effect of dexamethasone (DEX, 100 μM for 36 h) treatment on mTOR, p70S6K, and IGF-I expression in C2C12 cells. p-mTOR protein levels (A), p-p70S6K (B), and IGF-I mRNA level (C). The values are presented as the means ± SEM (n = 6). ^a,b^ Means with different letters differ significantly (*P <* 0.05).

We then investigated the effect of DEX treatment on the mTOR signalling pathway and on IGF-I expression. The results showed that the levels of phosphorylated mTOR and p70S6K proteins were both significantly decreased by DEX treatment (*P*< 0.05, Fig 4A and 4B). In addition, the mRNA level of IGF-I was downregulated by DEX treatment (*P*< 0.05, Fig 4C).

### Effect of myostatin blockage with follistatin on DEX-induced effects

**Fig 5:**
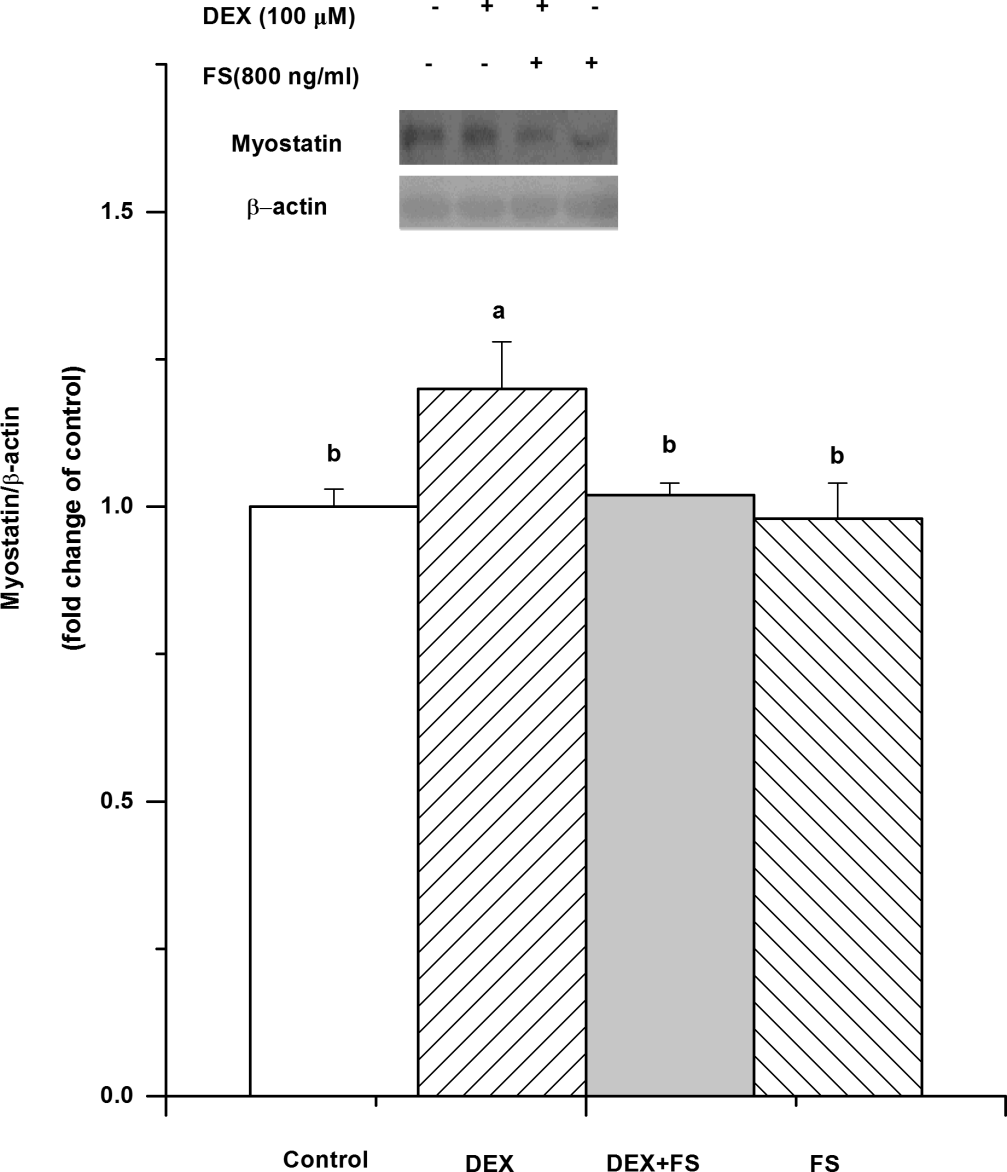
Effect of follistatin treatment on myostatin protein expression in C2C12 cells treated with dexamethasone (DEX, 100 μM for 36 h). The values are presented as the means ± SEM (n = 6). ^a,b^ Means with different letters differ significantly (*P <* 0.05).

To explore the effect of myostatin on protein metabolism, follistatin was used to inhibit myostatin in C2C12 cells. Follistatin treatment inhibited the DEX-induced increase of myostatin (*P*< 0.05, Fig 5). There was no significant difference between the control and follistatin treatments (*P*>0.05).

**Fig 6:**
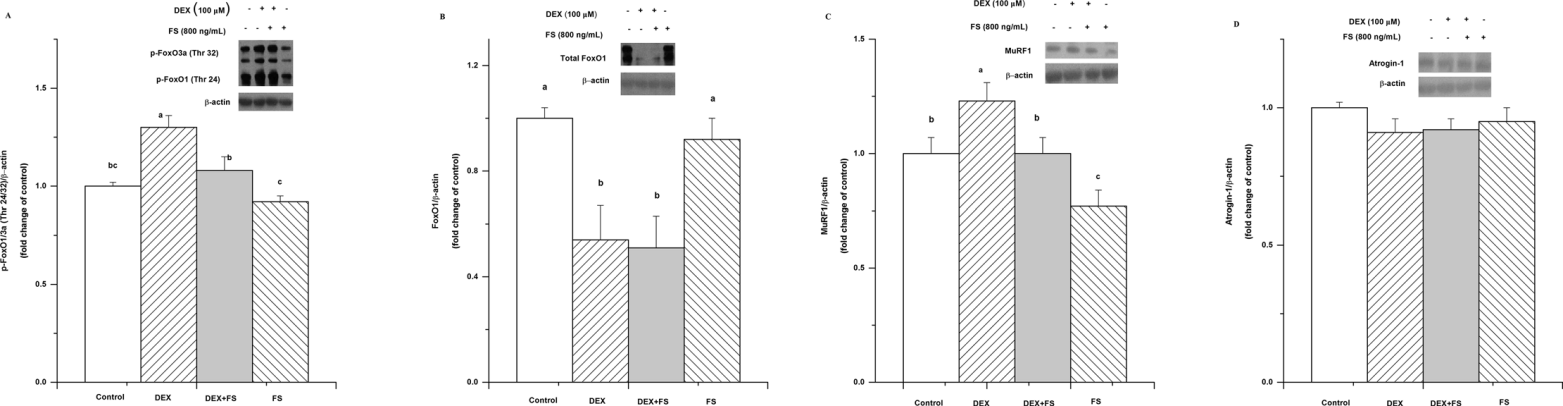
Effect of follistatin on the expression of ubiquitin-proteasome-related factors in C2C12 cells treated with dexamethasone (DEX, 100 μM for 36 h). The effect of DEX (100 μM) supplementation on C2C12 cells in the presence of follistatin (800 ng/ml) for 36 h on phospho-FoxO1/3a (Thr 24/32) (A), total FoxO1 (B), MuRF1 (C) and atrogin-1 (D) protein levels. The values are presented as the means ± SEM (n = 6). ^a,b^ Means with different letters differ significantly (*P <* 0.05).

Follistatin significantly inhibited the activation of phosphor-FoxO1/3a (Thr 24/32) (*P*< 0.05, Fig 6A) and MuRF1 (*P*< 0.05, Fig 6C,) caused by DEX. However, follistatin had no influence on the DEX-induced increases in total FoxO1 (*P*>0.05, Fig 6B) and atrogin-1 (*P*>0.05, Fig 6D).

**Fig 7:**
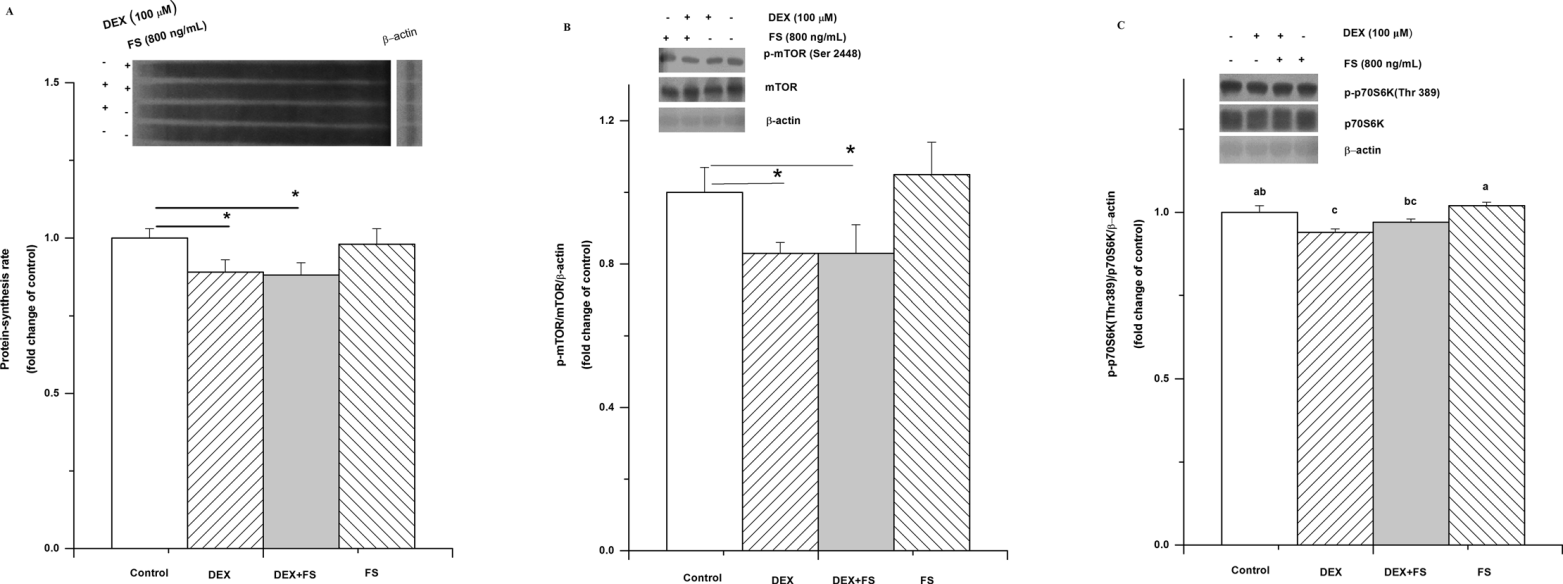
Effect of follistatin on protein synthesis and activation of mTOR/p70S6K pathway in C2C12 cells treated with dexamethasone (DEX, 100 μM for 36 h). The changes in protein synthesis rate (A) and phospho-mTOR (Ser 2448) (B) and phospho-p70S6K (C) levels in C2C12 cells after treatment with DEX (100 μM) and follistatin (800 ng/ml) for 36 h. The values are presented as the means ± SEM (n = 6). ^a,b^ Means with different letters differ significantly (*P <* 0.05).

Compared with synthesis in the control, follistatin had no significant influence on the protein synthesis rate (*P*>0.05, Fig 7A) or on p-mTOR (Ser 2448) (*P*> 0.05, Fig 7B) and p-p70S6K (Thr 389) (*P*> 0.05, Fig 7C) levels. Follistatin did not restore the suppressing effect of DEX on the protein synthesis rate or on the phosphorylation of mTOR and p70S6K (*P*>0.05).

## Discussion

In the present study, the role of myostatin in glucocorticoid-induced protein catabolism was investigated. DEX suppressed the protein synthesis rate and induced proteolysis. The inhibition of myostatin by follistatin attenuated the DEX-induced proteolysis by initiating the ubiquitin-proteasome system. These result suggests that myostatin is associated with the glucocorticoid-induced muscle protein catabolic effect rather than with the suppression of protein synthesis.

### Myostatin is involved in glucocorticoids (GCs)-induced muscle protein catabolism

In mammals, the GC-induced catabolic effect and muscle atrophy have been well studied [28]. Similar to the effects in mammals, GCs result in suppressed muscle development in chickens [29, 30]. In line with previous studies, the results of the present study show that the rate of protein synthesis was decreased by DEX treatment.

Myostatin is a member of the transforming growth factor β (TGF-β) member and is essential for the negative regulation of skeletal muscle growth [8]. Hence, we tested if myostatin was associated with the DEX-induced catabolic effect on skeletal muscle. In line with previous studies [31, 32], glucocorticoids upregulated both the mRNA level and protein level of myostatin. The present study showed that the protein level of myostatin was increased by DEX, suggesting that myostatin is associated with the catabolic effect induced by GCs. DEX-induced upregulation of myostatin mRNA was partially attributed to the binding of glucocorticoid receptor to glucocorticoid-binding element motifs along myostatin promoter [22]. In this study, the upregulated myostatin mRNA was observed at 24 h while myostatin protein at 36 h after DEX treatment indicated that the discrepancy between myostatin mRNA and protein levels. In C2C12 myoblast cells, addition of glutamine fully abolished the DEX-induced hyperexpression of myostatin at mRNA level rather than at protein level [21]. The posttranscriptional mechanism plays an important role in the regluation of myostatin [32]. Hence, the result implies that DEX could regulate myostatin expression at both transcriptional and posttranscriptional levels.

*In vivo*, myostatin mRNA and protein could be upregulated by dexamethasone at Day 5 and restored to normal level at Day 10 in a 10-day time course [31]. Recently, another research group reported that *in vivo* myostatin expression was upregulated by DEX at only Day 5 during 10-days treatment [33]. In line with the previous studies *in vivo*, the present *in vitro* result further demonstrated that dexamethasone induced upregulation of myostatin was time dependent. The result may suggest that myostatin play a role in glucocorticoid-induced muscle atrophy at the beginning of exposure.

Skeletal muscle atrophy is mediated through the activation of FoxO1 [34]. FoxO1 plays a important role in the regulation of atrogin-1 and MuRF1 expression in glucocorticoid induced muscle atrophy [7]. In human skeletal muscle tissue, the regulation of Akt and its downstream FoxO1 signalling pathway are both associated with the processes of skeletal muscle atrophy [35]. The proximal promoter of the FoxO1 gene contains multiple functional glucocorticoid response elements (GREs) and FoxO1 gene expression is regulated by binding of the glucocorticoid receptor to GREs [36]. We therefore measured the expression of FoxO1/3a and muscle atrophy-related genes, such as atrogin-1 and MuRF1. In line with previous study, FoxO1 mRNA was upregulated by DEX. DEX treatment regulated FoxO1 gene expression not only at the mRNA level but also at the protein level [36, 37]. The reduced phosphorylation is an important mechanism of FoxO1 activation [38]. In the present study, the p-FoxO1/3a was increased by DEX treatment, suggesting that the suppression of FoxO1 pathway, in line with the previous study [28]. However, the upregulated MuRF1 and antrogin-1 mRNA and MuRF1 protein by DEX indicated the activated muscle atrophy related genes, which agrees with previous results [28, 36, 37]. Recently, it was proved that the upregulation of MuRF1 in response to DEX might be mediated through the Kruppe-like factor 15 (KLF15) transcription factor, which is an Akt/FoxO-independent pathway [39]. Moreover, the transcription factor peroxisome proliferator-activated receptor β/δ (PPARβ/δ) upregulates muscle FoxO1 expression and activity with a downstream upregulation of atrogin-1 and MuRF1 expression during glucocorticoid [37]. Hence, the present implies that glucocorticoid could upregualte muscle atrophy related gene expression without the activation of FoxO1.

We thus hypothesized that myostatin may be involved in the GC-induced expression of MuRF1. Follistatin, a physiological inhibitor of myostatin, was used to reduce the effect of myostatin by binding to myostatin and acting as an effective myostatin inhibitor [40, 41, 42]. The suppression of protein expression caused by myostatin was eliminated by follistatin, suggesting that the effect of DEX was impeded by myostatin. Furthermore, the decrease in MuRF1 caused by follistatin further demonstrates that the GC-induced catabolic effect on muscle protein is at least partially via a myostatin-dependent pathway. Myostatin has been reported to be involved in stress-induced muscle atrophy. Acute daily psychological-stress-induced atrophic gene expression and the loss of muscle mass appear to be myostatin-dependent [2]. DEX-induced muscle loss is mediated, at least in part, by the upregulation of myostatin expression through a glucocorticoid receptor-mediated pathway [31]. The DEX-induced upregulation of myostatin gene expression was partly attributable to the binding of the glucocorticoid receptor to the glucocorticoid response element motifs in the myostatin promoter region [22]. Moreover, the increased level of phosphorylated FoxO1/3a caused by DEX was restored to normal by follistatin. The role of myostatin in the activation of Akt/FoxO1 needs to be investigated further.

### Myostatin is not responsible for the glucocorticoid-induced suppression of protein synthesis

High doses of GCs decrease the rate of protein accumulation by both increasing the rate of degradation and decreasing the rate of synthesis [4]. In rats, dexamethasone induces a significant decrease in protein synthesis in fast-twitch glycolytic and oxidative glycolytic muscles [43]. In line with previous studies, DEX decreased the protein synthesis rate in C2C12 cells.

The inhibitory effect of GCs on muscle protein synthesis is thought to result mainly from the inhibition of the mTOR/p70S6K pathway [44]. The Akt/mTOR pathway plays a role in the regulation of skeletal muscle hypertrophy [45]. Hence, we further investigated the effect of DEX on the mTOR pathway. The significant decreases in the phosphorylation level of mTOR and its downstream protein, S6K, indicate that the blockage of the mTOR/p70S6K pathway is involved in the DEX-induced suppression of protein synthesis. The activation of the Akt/mTOR pathway and its downstream targets, p70S6K and PHAS-1/4E-BP1, is essential in regulating skeletal muscle fibre size [46, 47, 48]. These results demonstrate that glucocorticoids could reduce the synthesis of proteins via the inhibitory effects of glucocorticoids on mTOR/p70S6K signalling.

Amirouche et al (2009) found that myostatin overexpression suppressed muscle protein synthesis by downregulating Akt/mTOR pathway [49]. We then investigated the role of myostatin in the reduction of protein synthesis by DEX. Although follistatin restored DEX-induced upregulation of myostatin (Fig 5), the inhibited protein synthesis or suppressed phosphorylation of mTOR and p70S6K induced by DEX were not eliminated by follistatin treatment, suggesting that myostatin was not a major factor contributing to the retarded protein synthesis induced by DEX.

Moreover, follistatin, besides of function as an inhibitor of myostatin, could mediate muscle growth independently of myostatin-driven mechanisms [50, 51]. Follistatin mediates *in vivo* muscle growth via Akt/mTOR/S6K signaling pathway [51]. Follistatin-induced muscle hypertrophy requires the activation of insulin/IGF-I pathway by either insulin or IGF-I [52]. In contrast, follistatin fails to stimulate muscle growth when both insulin and IGF-I are deficient [52]. In the present study, C2C12 were cultured with serum-free media to avoid the possible interference of serum. The absence of both insulin and IGF-I in the cultural media should be a reason of the unaffected mTOR/p70S6K in follostatin treatment. Therefore, the role of myostatin and follistatin in glucocorticoid-induced muscle development and growth remains to be elucidated with gene interfering technique in future [53].

In conclusion, the present study suggests that the myostatin signalling pathway is associated with glucocorticoid-induced muscle protein catabolism at the beginning of exposure and that myostatin may not a main pathway in the suppression of muscle protein synthesis by glucocorticoids.
